# The Mechanism of Action of Ethoxidol on Oxidative Stress Indices in Heart Failure and Hypotension

**DOI:** 10.17691/stm2020.12.2.08

**Published:** 2020

**Authors:** V.G. Kukes, O.K. Parfenova, B.K. Romanov, A.B. Prokofiev, E.V. Parfenova, N.G. Sidorov, A.A. Gazdanova, L.I. Pavlova, V.I. Zozina, A.D. Andreev, T.V. Aleksandrova, S.V. Chernova, G.V. Ramenskaya

**Affiliations:** Academician of the Russian Academy of Sciences, Professor, Department of Clinical Pharmacology and Propedeutics of Internal Diseases, I.M. Sechenov First Moscow State Medical University (Sechenov University), 8/2 Malaya Trubetskaya St., Moscow, 119991, Russia; Head of the Scientific Direction “Pharmacology”, Scientific Centre for Expert Evaluation of Medicinal Products of the Ministry of Health of the Russian Federation, 8, Bld. 2, Petrovsky Boulevard, Moscow, 127051, Russia;; Student, A.P. Nelyubin Institute of Pharmacy, I.M. Sechenov First Moscow State Medical University (Sechenov University), 8/2 Malaya Trubetskaya St., Moscow, 119991, Russia;; Deputy Director General on Scientific Work, Scientific Centre for Expert Evaluation of Medicinal Products of the Ministry of Health of the Russian Federation, 8, Bld. 2, Petrovsky Boulevard, Moscow, 127051, Russia;; Professor, Department of Clinical Pharmacology and Propedeutics of Internal Diseases, I.M. Sechenov First Moscow State Medical University (Sechenov University), 8/2 Malaya Trubetskaya St., Moscow, 119991, Russia; Director of the Clinical Pharmacology Center, Scientific Centre for Expert Evaluation of Medicinal Products of the Ministry of Health of the Russian Federation, 8, Bld. 2, Petrovsky Boulevard, Moscow, 127051, Russia;; Professor, Corresponding Member of the Russian Academy of Sciences, Deputy Director General, National Medical Research Center of Cardiology of the Ministry of Health of the Russian Federation, 15A 3^rd^ Cherepkovskaya St., Moscow, 121552, Russia; Director of the Institute of Experimental Cardiology, National Medical Research Center of Cardiology of the Ministry of Health of the Russian Federation, 15A 3^rd^ Cherepkovskaya St., Moscow, 121552, Russia;; Student, A.P. Nelyubin Institute of Pharmacy, I.M. Sechenov First Moscow State Medical University (Sechenov University), 8/2 Malaya Trubetskaya St., Moscow, 119991, Russia;; Associate Professor, Department of Clinical Pharmacology and Propedeutics of Internal Diseases, I.M. Sechenov First Moscow State Medical University (Sechenov University), 8/2 Malaya Trubetskaya St., Moscow, 119991, Russia;; Associate Professor, Department of Clinical Pharmacology and Propedeutics of Internal Diseases, I.M. Sechenov First Moscow State Medical University (Sechenov University), 8/2 Malaya Trubetskaya St., Moscow, 119991, Russia;; PhD Student, Department of Clinical Pharmacology and Propedeutics of Internal Diseases, I.M. Sechenov First Moscow State Medical University (Sechenov University), 8/2 Malaya Trubetskaya St., Moscow, 119991, Russia;; Student, Medical Faculty, I.M. Sechenov First Moscow State Medical University (Sechenov University), 8/2 Malaya Trubetskaya St., Moscow, 119991, Russia;; Senior Analyst, Scientific Centre for Expert Evaluation of Medicinal Products of the Ministry of Health of the Russian Federation, 8, Bld. 2, Petrovsky Boulevard, Moscow, 127051, Russia;; Associate Professor, Department of Pharmaceutical and Toxicological Chemistry named after A.P. Arzamastsev, I.M. Sechenov First Moscow State Medical University (Sechenov University), 8/2 Malaya Trubetskaya St., Moscow, 119991, Russia;; Director, A.P. Nelyubin Institute of Pharmacy, I.M. Sechenov First Moscow State Medical University (Sechenov University), 8/2 Malaya Trubetskaya St., Moscow, 119991, Russia; Head of the Department of Pharmaceutical and Toxicological Chemistry named after A.P. Arzamastsev, I.M. Sechenov First Moscow State Medical University (Sechenov University), 8/2 Malaya Trubetskaya St., Moscow, 119991, Russia

**Keywords:** active forms of oxygen, antioxidants, heart failure, hypertension, telomeres

## Abstract

**The aim of the investigation** was to study the effect of 2-ethyl-6-methyl-3-hydroxypyridine malate (Ethoxidol) on the concentration of oxidative stress metabolites in patients with chronic heart failure (CHF) and hypertension.

**Materials and Methods:**

126 patients with FC I–III CHF have been examined. In addition to their individual therapy these patients received intravenous infusions of Ethoxidol. Blood content of 2,3-diphosphoglycerate (2,3-DPG), oxygen tension (рО_2_), pH, concentration of total peroxides, lactate, and aldosterone were identified. 2,3-DPG levels (g/L erythrocytes) in whole blood samples were determined by an enzyme assay using the reagent kit (Rosh, Germany), values of рО_2_, рСО_2_, рН, lactate in the venous blood were measured using gas analyzer Stat Profil pHOx Ultra (Nova Biomedical, USA). Indices of oxidative stress, i.e. the concentration of plasma total peroxides, were investigated by ELISA using OxyStat kit (Biomedica, Austria). Peripheral venous blood samples were collected from all patients before and 6 days after the daily intravenous Ethoxidol infusion.

**Results:**

In patients with FC I, II, III CHF, on day 7 after intravenous Ethoxidol infusion at a dose of 100 mg/day, statistically significant growth (p=0.0002) of PaO_2_ level by 15.7, 17.4, and 22.8%, respectively, was noted. In patients with FC I, II, III CHF in the group receiving standard therapy, statistically significant (p=0.002) reduction of 2,3-DPG level by 2.7, 2.4, and 4.0%, respectively, was registered. On day 7 after the infusion of Ethoxidol at a dose of 100 mg/day, its decrease by 5.7, 10.5, and 26.2%, respectively (p<0.0001), was also observed.

**Conclusion:**

The increased concentrations of active oxygen forms have been established to negatively affect various bodily functions and adversely influence the pathophysiology of numerous diseases. Application of antioxidants, including Ethoxidol presented by us in this article, may become a clue to the development of preventive measures for many serious diseases.

## Introduction

Redox reactions underlying the metabolic processes in the human body result in the formation of peroxide compounds. In recent time, the effect of the active forms of oxygen (AFO) on the pathophysiology of numerous diseases is being studied and this interest is determined not only by their direct toxicity but the alteration of signal pathways which regulate functions of the cells and organs.

Organic peroxides are the first products of reactions taking place between the cell components and AFO. There is a direct relation between the presence of AFO (O./2, Н_2_О_2_) and the circulating biological peroxides. Active forms of oxygen are the necessary components of vital activity of the cells and the organism as a whole. They participate in many metabolic and regulatory processes. All these processes can be implemented only if the physiological level of AFO is maintained due to the functioning of antioxidant systems. For example, coenzyme Q10 takes part in the antioxidant protection of the organism [1–4]. Impairment in the work of the ubiquinone results in destabilization of the electron transport chain (ETC) that is one of the causes of myocardial contractility reduction. Antioxidants can also transport electrons and this contributes to normalization of the ETC work and improvement of myocardial contractility.

Oxidative stress is associated with free radicals and decreases the activity of enzymes and substances including 2,3-diphosphoglycerate (2,3-DPG). This secondary messenger located in erythrocytes influences their main function: oxygen transport. Changes in the 2,3-DPG content alter hemoglobin affinity to oxygen: accelerate dissociation of oxyhemoglobin into hemoglobin and oxygen. When 2,3-DPG content is reduced, oxygen tension (рО_2_) in the blood decreases. 2,3-DPG is completely blocked by Н_2_О_2_ [5–7]. In 2000, the authors [5] proved that if H_2_O_2_ is added, 2,3-DPG synthesis decreases, and in the works [6–8] it has been established to be blocked completely.

In hypoxia, the concentration of acidic products (lactate, active oxygen) is increased which causes the reduction of the activity of cytochrome P450 isoenzymes, for example, isoenzyme CYP 3A4, and this worsens the course of heart failure. CYP 3A4 is involved in the aldosterone metabolism, therefore, its activation increases the level of aldosterone metabolism [9] promoting the reduction of concentration and normalization of water-electrolyte metabolism [10].

Currently, a second-generation antioxidant, a low-toxic medical preparation 2-ethyl-6-methyl-3-hydroxypyridine malate (Ethoxidol), was registered in the Russian Federation. It causes antihypoxic effect in ischemic conditions developing during diabetes mellitus (a registered indication). The drug was synthetized and studied in 1993 by L.N. Sernov.

It has been established that in 10-month-old white rats with experimentally induced myocardial ischemia, Ethoxidol increased the enzymatic activity of the cellular antioxidant system, superoxide dismutase, and catalase, decreased the intensity of the oxidative stress, reduced the amount of lipid peroxidation products, diene conjugate, and malondialdehyde. All this is accompanied by the signs of cardiomyocyte membrane stabilization, decrease of the tissue hypoxia extent, and rise in the ATP content in the myocardial homogenate.

Based on the positive results of the preclinical investigations, a post-registration clinical assessment of the efficacy and safety of Ethoxidol extending its indications has been carried out [8].

**The aim of the investigation** was to study the mechanism of action of 2-ethyl-6-methyl-3-hydroxypyridine malate (Ethoxidol) on the concentration of oxidative stress metabolites in patients with chronic heart failure (CHF) and hypertension.

## Materials and Methods

126 patients with CHF, I–III functional class (FC), participated in the study. They received intravenous infusions of Ethoxidol additionally to their individual therapy. The study complied with the Declaration of Helsinki (2013) and was approved by the Ethical Committee of I.M. Sechenov First Moscow State Medical University. Written informed consent was obtained from every patient.

Blood content of 2,3-DPG, рО_2_, pH, concentration of total peroxides, lactate, and aldosterone were identified. 2,3 DPG levels (g/L erythrocytes) in whole blood samples were determined by an enzyme assay using the reagent kit (Rosh, Germany), values of рО_2_, рСО_2_, рН, lactate in the venous blood were measured using gas analyzer Stat Profil pHOx Ultra (Nova Biomedical, USA). Indices of oxidative stress, i.e. the concentration of plasma total peroxides, were investigated by ELISA using OxyStat kit (Biomedica, Austria). Peripheral venous blood samples were collected from all patients before and 6 days after the daily intravenous Ethoxidol infusion.

**Statistical data processing.** The results were analyzed using IBM SPSS Statistics 20.0 program. To describe the values presented in the quantitative variables at the assumed normal distribution of the general population, parametric methods of descriptive statistics were used: sample average ± standard deviation (x±σ). Differences were considered significant at p<0.05.

## Results

In patients with FC I, II, III CHF, a statistically significant growth (p=0.0002) of PaO_2_ level by 15.7, 17.4, and 22.8%, respectively, was noted at day 7 after intravenous Ethoxidol infusion at a dose of 100 mg/ day (see the Table). When assessing the efficacy of the standard therapy (before Ethoxidol infusion) in the patients with FC I, II, III CHF, statistically significant (p=0.002) reduction of 2,3-DPG level by 2.7, 2.4, and 4.0%, respectively, was registered. On day 7 after the infusion of Ethoxidol at a dose of 100 mg/day, a more considerable statistically significant (p<0.0001) decrease by 5.7, 10.5, and 26.2%, respectively, was observed.

**Table:** Dynamics of indices in patients with chronic heart failure before and after treatment with Ethoxidol (х±σ)

Indices	Standard therapy	After treatment with Ethoxidol
pO2 (mm Hg)	39.26±16.80	59.96±23.27
2,3-DPG (g/L):		
FC I, II	0.51±0.04	0.43±0.04
FC III	0.23±0.02	0.32±0.02
EchoCG, ejection fraction (%)	51.60±5.88	53.40±5.45
Total concentration of organic peroxides (μmol/L)	1220±250	1050±210
pH	7.41±0.04	7.39±0.04
Lactate (mmol/L)	2.23±0.64	1.91±0.76
Diuresis volume (ml)	490.0±188.40	1020.0±178.80

## Discussion

The obtained data allow us to suppose that Ethoxidol action depends on the feedback mechanism of the oxidase systems, that is on the increase of the superoxide dismutase (SOD) activity. It may be connected with the increase of O./2 production which subsequently reduces the concentration of organic peroxides [11].

Sousa et al. [12] have studied the pathogenesis of hypertension. Since angiotensin II (Ang II) is the main hormone rising blood pressure, they injected it to the rats in order to determine the ways of hypertension development. This hormone causes the efferent arterioles to constrict and, consequently, there occurs the reduction of hydrostatic and increase of oncotic pressure in peritubular capillaries — both of these effects result in the increase of water and sodium reabsorption. To evaluate the effect of the treatment, catalase-polyethylene glycol (PEG-catalase) was injected to the rats.

**Figure 1:**
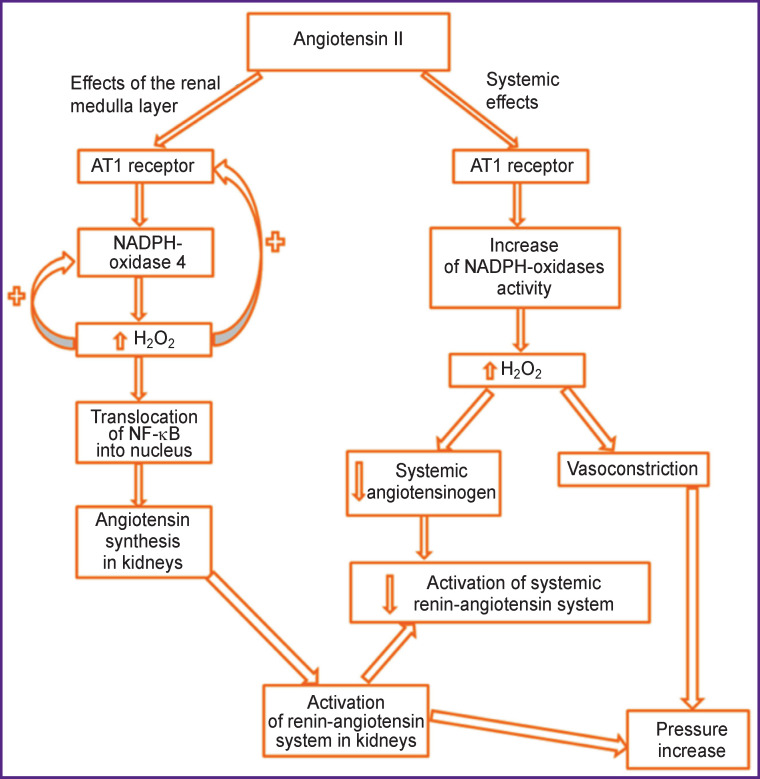
Mechanisms involved in the elevation of arterial pressure and activation of renin-angiotensin system by means of Ang II and Н_2_О_2_ [12]

Expression of H_2_O_2_ was increased with Ang II injection. The Н_2_О_2_ level was higher in blood plasma and urine, however, Ang II caused a double effect on the angiotensinogen level: it became higher in urine but lower in plasma. Besides, Ang II raised the Н_2_О_2_ level in the renal medulla layer whereas PEG-catalase reduced it (Figure 1).

In the rats with hypertension, the rise of Н_2_О_2_ systemic level has been established in urine as well which causes narrowing of the vessels and pressure elevation.

The elevation of the Н_2_О_2_ level increases Ang II synthesis in kidneys which contributes to the activation of renin-angiotensin system and, consequently, to the pressure elevation. PEG-catalase produced only a short-time effect despite a steady reduction of Н_2_О_2_ level [12]. Though arterial pressure was noticeably reduced during the first days of PEG-catalase injection, this effect was not observed at the end of the treatment, which signified the necessity of antioxidant application.

Münzel et al. [13] have considered the molecular basis of the oxidative stress in heart failure. It is characterized by the activation of the sympathetic nervous and renin-angiotensin-aldosterone systems. This neuroendocrine activation is connected with the oxidative stress in the myocardium and vascular network. In patients with heart failure, oxidative stress occurs in the myocardium and blood plasma and correlates with left ventricle dysfunction. AFO affect negatively calcium transfer in the myocardium (Ca^2+^) that causes arrhythmia and promotes heart remodeling inducing a hypertrophic signal transmission, apoptosis, and necrosis. Enzymatic sources for AFO such as NADPH-oxidases (NOX), unbound nitric oxide (NO), and mitochondria are considered the sources of AFO in heart failure causing dysfunction of the vessels and myocardium. It is important that mitochondria enhance the synthesis of AFO originating from NOX and can in this way function as redox centers in the physiology of the heart and in its illness [13].

In myocytic mitochondria, O_2_ is generated by the ETC, but is quickly changed into Н_2_О_2_ by manganese-dependent SOD. Н_2_О_2_ is then eliminated by the antioxidant enzymes (glutathione peroxidase and peroxiredoxin) which generate NADPH [13]. In mitochondria, the Krebs cycle generates NADH, which yields electrons to ETC for ATP production. Nevertheless, the Krebs cycle also produces substrates restoring NADPH, which, in its turn, regenerates antioxidant enzymes. In heart failure, defects in the location of cytosolic Ca^2+^ and Na^+^ in cardiac myocytes (e.g. decrease in the release of sarcoplasmic reticulum Ca^2+^ and elevation of Na^+^) diminish the accumulation of mitochondrial Ca^2+^ and thereby prevent regeneration of NADH and NADPH interrupting the ATP production by provoking AFO ejection from mitochondria [13, 14].

In heart failure, the limited substrates for ATP production (i.e. NADH) and AFO elimination (i.e. NADPH) face the demand in energy caused by a high cardiac preload, afterload, and heart rate. In heart failure, the increased AFO production and reduction in their elimination in the cardiac myocytes promote the increase of pure ejection of mitochondrial AFO which play the main role in the pathogenesis of heart failure (decrease of antioxidant enzyme concentrations) [13].

Active forms of oxygen regulate numerous cellular functions including growth and proliferation of endothelial and smooth muscle cells. However, extreme levels of oxidants mediate vascular diseases via direct and irreversible damage of macromolecules and growth of the redox potential in the vascular walls as well [13, 14].

In the vascular network, O_2_ is generated by NADPH-oxidase, xanthine oxidase, and mitochondria. SOD converts O_2_ into Н_2_О_2_. Due to the Fenton reaction, Н_2_О_2_ can spontaneously change into a hydroxyl radical OH. Being highly reactive OH can damage the majority of cellular compartments [13].

**Figure 2:**
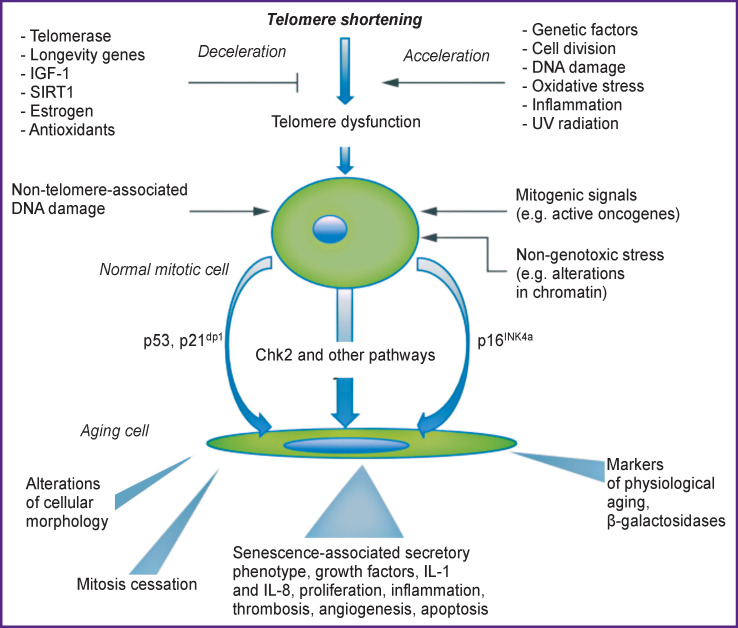
Factors influencing the telomere length [15]

Formation of AFO and oxidative stress mediate injury of tissues and cells, which may turn into an inflammation cycle. Investigations have shown that AFO may accelerate telomere shortening and damage the DNA, and thereby induce aging. Oxidants cause telomere attrition in the cultured human endothelial cells whereas antioxidants prevent their shortening. Aging, in its turn, results in further AFO generation. Telomere dysfunction and vascular aging are associated with the increased generation of AFO, molecules of adhesion and inflammation, and also beta-galactosidase. Cell division and telomere DNA damage are the main factors leading to telomere shortening and dysfunction. The oxidative stress and inflammation promote strongly telomere attrition resulting in cell aging. And vice versa, in the vicious circle, cellular aging induces inflammation and formation of oxidative radicals [15] (Figure 2).

**Figure 3:**
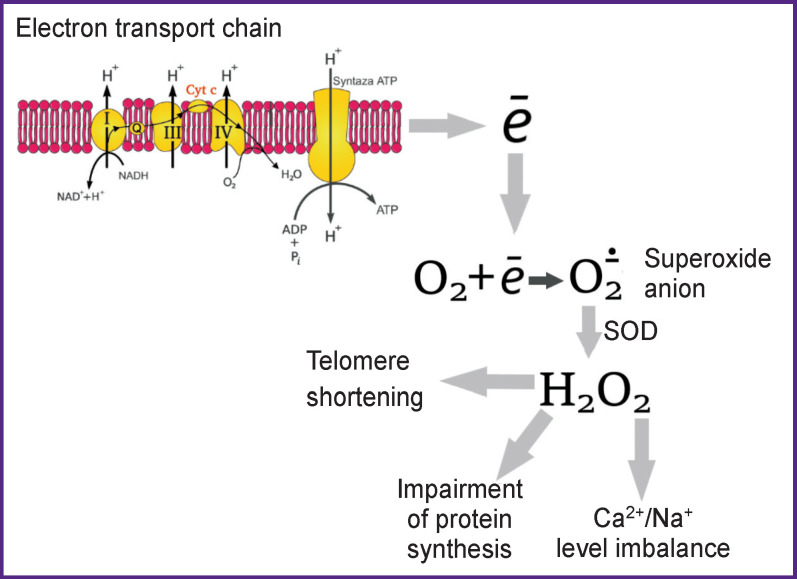
Negative Н_2_О_2_ manifestations

Thus, a negative effect of AFO causes progression of heart failure, therefore, the application of antioxidants directed to the blockage and inactivation of their action is quite reasonable. In the mentioned Russian and foreign investigations, an important role of these forms in the cellular metabolism is noted, but at the same time, a negative effect of the increased AFO concentration, especially that of Н_2_О_2_, on various body functions is indicated as well as a negative influence on the pathophysiology of numerous diseases (Figure 3).

## Conclusion

The increased concentrations of active forms of oxygen have been established to negatively affect various bodily functions and adversely influence the pathophysiology of numerous diseases. Application of antioxidants, including Ethoxidol presented by us in this article, may become a clue to the development of preventive measures for many serious diseases.
